# Real-Time PPP Based on the Coupling Estimation of Clock Bias and Orbit Error with Broadcast Ephemeris

**DOI:** 10.3390/s150717808

**Published:** 2015-07-22

**Authors:** Shuguo Pan, Weirong Chen, Xiaodong Jin, Xiaofei Shi, Fan He

**Affiliations:** 1 School of Instrument Science and Engineering, Southeast University, Nanjing 210096, China; 2 School of Transportation, Southeast University, Nanjing 210096, China; E-Mails: cwr@seu.edu.cn (W.C.); jxd134@seu.edu.cn (X.J.); shixiaofei@seu.edu.cn (X.S.); hf117@seu.edu.cn (F.H.)

**Keywords:** clock estimation, precise point positioning, orbit error, CORS network

## Abstract

Satellite orbit error and clock bias are the keys to precise point positioning (PPP). The traditional PPP algorithm requires precise satellite products based on worldwide permanent reference stations. Such an algorithm requires considerable work and hardly achieves real-time performance. However, real-time positioning service will be the dominant mode in the future. IGS is providing such an operational service (RTS) and there are also commercial systems like Trimble RTX in operation. On the basis of the regional Continuous Operational Reference System (CORS), a real-time PPP algorithm is proposed to apply the coupling estimation of clock bias and orbit error. The projection of orbit error onto the satellite-receiver range has the same effects on positioning accuracy with clock bias. Therefore, in satellite clock estimation, part of the orbit error can be absorbed by the clock bias and the effects of residual orbit error on positioning accuracy can be weakened by the evenly distributed satellite geometry. In consideration of the simple structure of pseudorange equations and the high precision of carrier-phase equations, the clock bias estimation method coupled with orbit error is also improved. Rovers obtain PPP results by receiving broadcast ephemeris and real-time satellite clock bias coupled with orbit error. By applying the proposed algorithm, the precise orbit products provided by GNSS analysis centers are rendered no longer necessary. On the basis of previous theoretical analysis, a real-time PPP system was developed. Some experiments were then designed to verify this algorithm. Experimental results show that the newly proposed approach performs better than the traditional PPP based on International GNSS Service (IGS) real-time products. The positioning accuracies of the rovers inside and outside the network are improved by 38.8% and 36.1%, respectively. The PPP convergence speeds are improved by up to 61.4% and 65.9%. The new approach can change the traditional PPP mode because of its advantages of independence, high positioning precision, and real-time performance. It could be an alternative solution for regional positioning service before global PPP service comes into operation.

## 1. Introduction

Precise point positioning (PPP) is a cutting-edge theory and a significant topic in the field of Global Navigation Satellite System (GNSS) navigation and positioning. PPP technology uses the corrections of the parameter field to realize precise positioning through a dual-frequency receiver at any position in International Terrestrial Reference Frame (ITRF) [1,2,3,4]. The realization of this technology can change the status of obtaining high-precision location information, which only relies on double-difference mode. This technology can prevent the regional restriction caused by the relative positioning of ground reference stations and has important and broad application prospects. In the process of PPP calculation, satellite orbit error and satellite clock bias cannot be corrected by empirical models or eliminated through station difference. Therefore, satellite orbit error and clock bias need to be calculated on the basis of the observations of a ground-tracking station network. The error can directly affect PPP resolving precision. These factors are the critical and core issues of achieving PPP.

Traditional PPP studies often use third-party precise orbit and clock products for PPP, such as the current most widely used precise satellite orbit and clock products provided by the International GNSS Service (IGS) [5,6]. The IGS final products can achieve 2 cm accuracy for orbit and 0.075 ns accuracy for clock [7]. However, real-time positioning services will be the dominant mode in the future [8,9]. IGS is providing such an operational service (RTS) and there are also commercial systems like Trimble RTX in operation [10,11,12]. The real-time product can achieve 2 cm accuracy for orbit and 0.3 ns accuracy for clock [13,14]. These products usually have a significant time delay with a fixed sampling interval. The final precise IGS ephemeris is usually available after 12–18 days. Rapid ephemeris is available after 17–41 h, and the observed half of the ultra-rapid ephemeris is available after 3–9 h. In the latest real-time data stream products provided by IGS, the orbit and clock products have a sampling interval of approximately 30 s. However, the direct use of such products cannot meet the needs of real-time PPP. The fixed sampling interval of these products also cannot meet the application needs of travel, aircraft carriers, and other high sampling rate. To solve the abovementioned problems, Gao *et al.* [15] and Ge *et al.* [16] applied a simple interpolation process for satellite orbit, with an extrapolation forecast of several hours, which can guarantee the orbit accuracy. The linear interpolation method would incur serious accuracy loss because of the discrete nature of satellite clock error, which is the key problem of the current real-time PPP. The linear interpolation for satellite clock bias with 5 min sampling interval cannot meet the cm-level orbit determination for Low Earth Orbit (LEO) satellites in PPP [17]. Furthermore, IGS reference stations belong to more than 100 research institutions, universities, and government organizations; thus, data coordination is difficult. The practice requirement is also difficult to protect. Overall, PPP is currently relatively mature for post-processing. Nevertheless, real-time positioning is still difficult to conduct in consideration of the requirement of precise orbit and clock products provided by the GNSS analysis center.

Since third-party precise orbit and clock error products are inadequate for real-time PPP, Yan *et al.* [18] and Ge *et al.* [19] applied a method using regional Continuous Operational Reference System (CORS) to estimate orbit and clock error. The method is based on ground-tracking station networks covering a region. Each station transmits real-time observation data to the system control center. Thus, satellite orbit and clock are precisely determined to provide real-time PPP service for regional users. Although the use of regional CORS can effectively estimate satellite clock error, very small-scale regional reference station network cannot be used to determine precise satellite orbit because orbit determination is restricted by the continuous arc observation time. This method has claim on the size of CORS network. A large CORS network contains many ground-tracking stations, thus increasing time needed to estimate unknown parameters. In addition to the satellite orbit and clock error, the estimated parameters also contain phase ambiguity, receiver clock error, station zenith tropospheric delay, *etc*. The huge computational load is a severe test for the real-time computing and external broadcast capability of a system. Therefore, some improvements are made for real-time PPP [20,21,22]. The real-time clock bias is estimated on the basis of known precise satellite orbits. The observations from regional CORS and the predicted IGS ultra-rapid orbit are applied to estimate the satellite clock bias. The PPP service can then cover a larger area than the CORS service. This method does not need to determine the satellite orbit autonomously, and the system is relatively simple to realize. Hence, this method is the most common way for achieving real-time PPP processing in small areas, such as Chinese provincial and municipal reference station networks. In the case of a CORS network using low-cost, single-frequency receivers, uncombined GNSS data plus ionospheric delays from the CORS is a better choice for PPP [23]. The uncombined algorithm can avoid the effects of amplified observational noise and multipath effects, as well as possible information lost [24]. However, the system needs to receive real-time IGS satellite orbit products. The system is also not a truly rigorous independent real-time PPP algorithm because the operation of the system is affected by the real-time performance and service quality of the IGS satellite orbit.

When utilizing the satellite orbit and clock error products offered by IGS or using regional CORS to autonomously estimate clock error, the orbit error and clock error need to be estimated. However, judging from the ultimately positioning demands of PPP, in consideration of the positioning results only, the satellite orbit and clock error do not need to be distinguished. We can couple the clock and orbit error to estimate and obtain a clock error product that absorbs some of the broadcast ephemeris orbit errors. When turning to broadcast ephemeris for positioning, the technical limitations of third-party precise ephemeris for real-time PPP can be avoided.

We find that the satellite orbit error and clock bias have an approximately consistent direction, thus inspiring us to couple the satellite orbit and clock bias as a parameter to estimate. We can then realize PPP completely and autonomously by using the broadcast ephemeris to couple the estimation of orbit and clock errors through regional CORS.

The remainder of this article is organized as follows. Section 2 presents the algorithm model for simultaneously estimating satellite clock error and orbit error, which is derived theoretically and is verified. Section 3 provides the designed system based on the coupling estimation of the clock and orbit errors on real-time PPP. Section 4 shows the measured data from different ways to verify the feasibility of the newly proposed algorithm and be compared with IGS real-time data stream products on PPP in terms of accuracy, convergence time, and other indicators. Section 5 summarizes the characteristics and advantages of the new method and provides the conclusions.

## 2. Estimation for the Parameter of Coupling Clock Bias with Orbit Error (PCCO)

### 2.1. Coupling of Clock Bias and Orbit Error

Broadcast ephemeris is commonly used in real-time positioning because of its easy acquisition and real-time availability. However, its application is limited by orbit precision. The orbit error of broadcast ephemeris can be divided into the radial part (R-orbit error), along-track part (A-orbit error), and the cross-track part (C-orbit error) in the satellite coordinate system. The broadcast ephemeris on 8 August 2013 is applied for orbit error analysis. The precise ephemeris provided by National Geospatial-intelligence Agency (NGA) is treated as the true value. The result from the broadcast ephemeris is the position of the satellite antenna phase center, whereas the precise ephemeris gives the position of satellite mass center [25]. Therefore the satellite antenna-phase center offset should be corrected before comparison. The satellite position from broadcast ephemeris is compared with the NGA orbit products with the antenna-phase center offset corrected. Since the precise ephemeris has a large fixed sampling interval, the comparison is only conducted every 15 min to avoid interpolation error.

**Figure 1:**
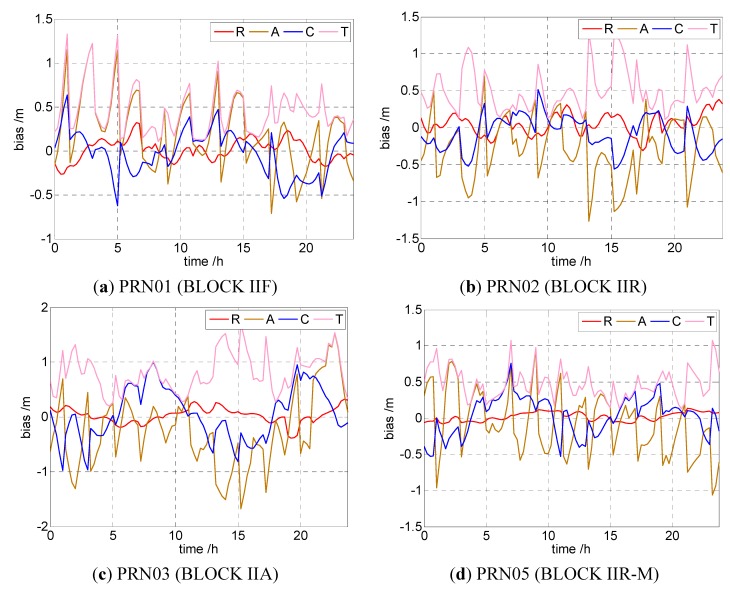
Orbit error statistics of the satellites with different types calculated from broadcast ephemeris. (**a**) BLOCK IIF; (**b**) BLOCK IIR; (**c**) BLOCK IIA; (**d**) BLOCK IIR-M.

By calculating the three components of the orbit error from broadcast ephemeris, we find some stochastic characteristics for each component. Four satellites with different types (BLOCK IIF, IIR, IIA, and IIR-M) are chosen as an example. Figure 1 shows the orbit error of broadcast ephemeris in the Radial (R), Along (A), and Cross (C) directions, and the Total (T) error. The total orbit error is calculated as follows:

(1) $$\text{Δ}s\text{=}\sqrt{\text{Δ}s_{R}^{2}+\text{Δ}s_{A}^{2}+\text{Δ}s_{C}^{2}}$$

where $\text{Δ}s_{R}$, $\text{Δ}s_{A}$, and $\text{Δ}s_{C}$ represent the orbit errors in the radial direction, along-track direction, and cross-track direction, respectively; and Δs represents the total orbit error.

From Figure 1 we can see that the total orbit error of broadcast ephemeris is about 1 m. All three components of orbit error fluctuate near the zero mean value. The bias in the radial is rather smaller than the other two components. However, considering that the orbit error is important for receiver positioning, even the small R-orbit error must be processed for PPP. The correlation between the satellite clock bias and orbit error is analyzed.

The GPS dual-frequency ionosphere-free observation equations are written as follows:

(2) $$\begin{array}{c} \text{Δ}P_{IF}=-\text{μ}\cdot \text{Δ}r+\text{μ}\cdot \text{Δ}s+cdt^{r}-cdt^{s}+M\cdot zpd+{\text{ε}}_{P_{IF}}\\ \text{Δ}L_{IF}=-\text{μ}\cdot \text{Δ}r+\text{μ}\cdot \text{Δ}s+cdt^{r}-cdt^{s}+M\cdot zpd+A_{IF}+{\text{ε}}_{L_{IF}} \end{array}$$

where $\text{Δ}P_{IF}$ and $\text{Δ}L_{IF}$ denote the ionosphere-free pseudorange observation residual and the ionosphere-free phase observation residual, respectively; $A_{IF}$ denotes the ionosphere-free real-valued ambiguity; μ denotes the unit vector of the satellite to the station; Δr and Δs denote the coordinate corrections of the station and satellite, respectively; $cdt^{r}$ and $cdt^{s}$ denote the receiver clock offset and the satellite clock error, respectively; *M* denotes the troposphere mapping function; *zpd* denotes the zenith tropospheric delay; and ${\text{ε}}_{P_{IF}}$ and ${\text{ε}}_{L_{IF}}$ denote the multipath and other measurement noises, respectively.

**Figure 2:**
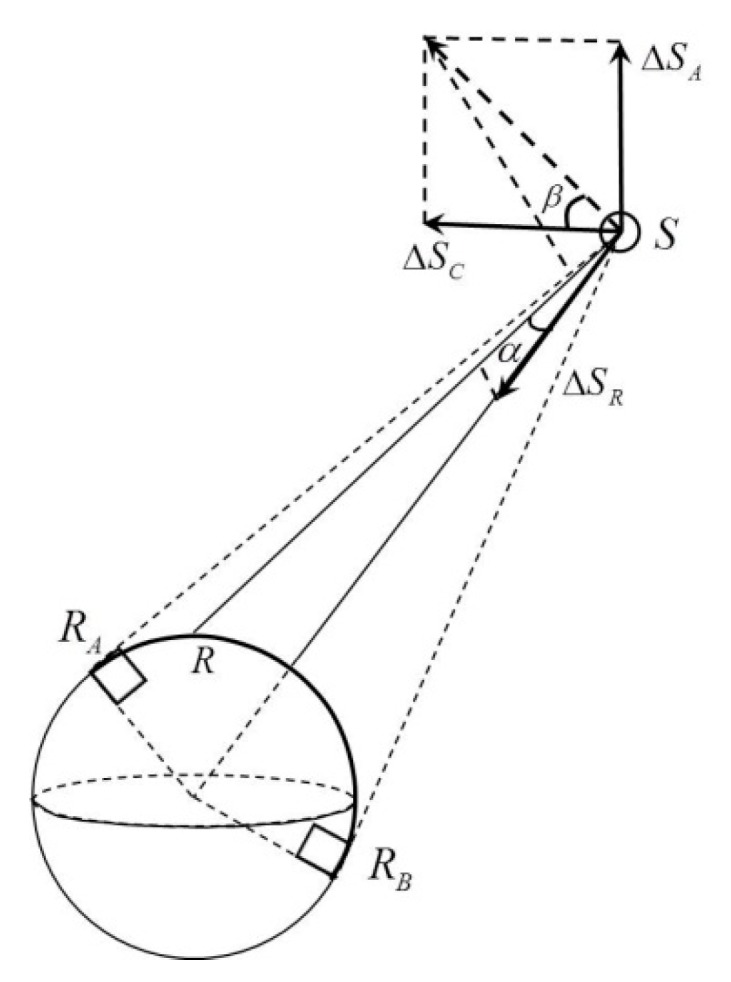
The geometry relationship between the orbit error (radial, along, and cross direction) and the satellite to receiver direction. (R: rover; R_A_ and R_B_: rover position with altitude angle of zero; S: satellite).

In traditional PPP, the precise ephemeris and satellite clock bias provided by IGS are already known. The unknown parameters include the receiver coordinate corrections, receiver clock bias, zenith tropospheric delay, and real-valued ambiguities. If broadcast ephemeris is applied to replace the precise ephemeris, the orbit error in meter size of broadcast ephemeris will be introduced. Therefore, the orbit error of broadcast ephemeris cannot be ignored. The geometry relationship between the orbit error and satellite to receiver direction is shown in Figure 2.

From Figure 2 we can see that, to track a satellite, the receiver on the ground could just move in the radian R_A_R_B_ (2D geometry). According to the definition of radial direction, it is perpendicular to the direction of satellite movement in the orbital plane. If the satellite moves in a circular pattern, the radial direction points to the Earth’s center. For GPS satellites, the satellite orbit can be determined as a circle (only with elasticity of 0.01); thus, the radial direction can be approximated as the direction from the satellite to the Earth’s center. The angle between the satellite vector to the station and the satellite vector to the Earth’s center can be expressed as α. As shown in Figure 2, the maximum value of α can be calculated as follows:

(3) $${\text{α}}_{\text{max}}=\text{arcsin}\left(\frac{R}{R+H}\right)$$

where *R* denotes the radius of the Earth and *H* denotes the height of the satellite orbit. The orbital altitude of the GPS satellite orbit is approximately 20200 km. Hence, if a user on Earth can receive the signal of GPS satellites, the maximum value of α will be 13.9°.

When it comes to the along and cross direction, the projection is determined not only by angle α, but also the angle between the cross direction and the plane defined by satellite, receiver, and the Earth’s center. The angle can be expressed as β. Without thinking about the surface configuration, we assume that the receiver could move to anywhere on the ground; β changes from −90° to 90°.

Therefore, the projection onto satellite to receiver range of the satellite orbit error can be calculated as follows:

(4) $$\text{Δ}=\text{Δ}s_{R}\cdot \cos\text{α}+\left(\text{Δ}s_{A}\cdot \sin\text{β}+\text{Δ}s_{C}\cdot \cos\text{β}\right)\cdot \sin\text{α}$$

where Δ is the projection in the direction of the satellite to the receiver. Since α is between 0° and 13.9°, cosα∈(0.971,1] and sinα∈(0,0.240]. By setting a certain cut-off angle in the receiver, cosα value can be closer to one and sinα value can be closer to zero. Thus, most of the R-orbit error can be projected onto the satellite-receiver range, while the A-orbit error and C-orbit error cannot be projected so far.

The satellite clock bias can be viewed in terms of distance measured in the direction of the satellite to the receiver. Therefore, the orbit error can be absorbed during clock bias estimation. Lou *et al.* [26] have verified that, considering that all visible satellites are evenly distributed in the sky above the station, a part of the remaining unabsorbed orbit errors of several satellites can offset one another in the unified calculating process. The effect caused by the residual orbit error can then be weakened by PPP.

### 2.2. Joint Weighted Estimation of PCCO Based on Regional CORS

The regional reference stations have advantages, such as precise coordinates of stations already known, continuous operation, high observation data quality, and convenient observation data acquisition. These advantages provide a good platform for real-time satellite clock bias estimation. On the basis of Equation (2), the single difference between satellites is applied to estimate the satellite clock. The observations are as follows:

(5) ΔPIFi,j=Mi,j⋅zpd−c(dti,j−Δi,jc)+εi,j(PIF)ΔLIFi,j=Mi,j⋅zpd+AIFi,j−c(dti,j−Δi,jc)+εi,j(LIF)

where *i* and *j* denote the reference and non-reference satellites, respectively. The other parameters are the same as in Equation (2). By using the single difference between satellites, the receive clock bias is eliminated. The precise station and satellite coordinates are already known. Thus, Equation (5) is more simplified than Equation (2). In particular, dti,j−Δi,jc denotes the satellite clock bias that absorbs the satellite orbit error. In this way, this satellite clock bias is no longer the same as that provided by IGS. According to Equation (5), satellite clock bias and phase ambiguity are the main parts of the unknown parameters. Since these two parameters have the same quantity and coefficient, a strong correlation exists between them. More than 20 min of observation time is usually needed to separate the two parameters by using the traditional method. To overcome this defect, we promote a modified satellite clock bias estimation method as follows:

1. At the first epoch, the satellite clock bias is calculated by using the pseudorange observation equation;
2. At epoch n, through the difference among the phase observations, the epoch differential phase observations are added to the pseudorange observation and the initial epoch to create a new pseudorange observation ${\bar{P}}_{IF,n}^{i,j}$. The expression is expressed as follows:
  (6) $${\bar{P}}_{IF,n}^{i,j}=P_{IF,0}^{i,j}+(L_{IF,n}^{i,j}-L_{IF,0}^{i,j})$$

The calculation model of the satellite clock bias estimation with smoothing pseudorange can be written as follows:

(7) $$dt^{i,j}=(M^{i,j}\cdot zpd+\text{ε}(L_{IF,n}^{i,j})-\text{Δ}{\bar{P}}_{IF,n}^{i,j})/c$$

A deviation is introduced to the satellite clock bias by the pseudorange observation noise in the initial epoch for any satellites. The deviation in the satellite clock bias belongs to the systemic error in the estimation process. Therefore, such a deviation can be absorbed by the ambiguity parameters and does not affect the positioning result. This clock bias estimation method is similar to the traditional method. The traditional method applies epoch differential phase observations to eliminate the ambiguity parameters. In the process of satellite clock bias estimation, the variation in zenith tropospheric delay can be considered the unknown parameters for estimation. The traditional method uses empirical models to correct zenith tropospheric delay.

During the estimation processing of satellite clock bias in the regional reference station network, the troposphere mapping function of the same satellite at different sites is similar. Hence, unified estimation with several stations will cause a strong correlation among the zenith tropospheric delays. For this reason, the satellite clock bias is estimated in each station. The weighted average is calculated from each satellite clock bias $\text{Δ}t_{i,m}^{s}$. For example, in epoch *i*, the weight *k* of satellite *s* in station *m* is calculated, and the weighted clock bias $\text{Δ}t_{i}^{s}$ is as follows:

(8) $$\{\begin{array}{c} k_{i,m}^{s}=1/(\sqrt{\frac{1}{{\text{sin}}^{2}(E_{i,m}^{s})}+\frac{1}{{\text{sin}}^{2}(E_{i,m}^{ref})}})\\ \text{Δ}t_{i}^{s}=(k_{i,1}^{s}\cdot \text{Δ}t_{i,1}^{s}+k_{i,2}^{s}\cdot \text{Δ}t_{i,2}^{s}\cdots k_{i,N}^{s}\cdot \text{Δ}t_{i,N}^{s})/\sum_{m=1}^{N}k_{i,m}^{s} \end{array}$$

where *E* denotes the elevation of satellite.

The improved method for satellite clock bias estimation (including orbit error) does not need to consider the effect of ambiguity parameters. With each station independently calculating satellite clock bias, the final real-time satellite clock product could be obtained through the elevation weighted average. This method can reduce the overload of satellite clock bias calculation and provides the satellite clock bias immediately.

### 2.3. PCCO Effect on Observation Equations

According to the previous analysis, most of the R-orbit error and part of the A-orbit error and C-orbit error from the broadcast ephemeris can be absorbed by satellite clock bias. The residual orbit error is weakened or even offset by its own random characteristics and the unified calculation of satellites at different azimuths. The influence of satellite clock bias that absorbs part of the orbit error is determined by analyzing the Observed Minus Computed (OMC) value during rover positioning.

The dual-frequency ionosphere-free observation equation can be transformed from Equation (2). The detailed information is expressed as follows:

(9) $$\text{Δ}L_{IF}+cdt^{s}-\text{μ}\cdot \text{Δ}s=-\text{μ}\cdot \text{Δ}r+cdt^{r}+M\cdot zpd+A_{IF}+{\text{ε}}_{L_{IF}}$$

where the observation residual $\text{Δ}L_{IF}$ is already known and satellite clock bias $dt^{s}$ is estimated and sent to the user by CORS center. Since the satellite position calculated by broadcast ephemeris is considered as the precise value, the satellite orbit error Δs can be ignored. In this way, the value to the left of the equals sign is expressed as $L_{\Phi }$. Thus, the formula can be expressed as follows:

(10) $$L_{\Phi }=AX+{\text{ε}}_{L_{IF}}$$

where *A* denotes the coefficient matrix and $X={\left[\text{Δ}r,dt^{r},zpd,A_{IF}\right]}^{T}$ denotes the unknown parameter vector. For convenience of calculation, the difference between satellites is applied to eliminate the receiver clock bias. The OMC vector of observation equation could be obtained as follows:

(11) $$L=L_{\Phi }^{rover}-L_{\Phi }^{pivot}$$

where $L_{\Phi }^{rover}$ denotes the OMC vector of rover satellite-phase observation and $L_{\Phi }^{pivot}$ denotes the OMC vector of pivot satellite-phase observation.

The comparison of the traditional PPP methods and PCCO methods in real-time PPP indicates that the coefficient matrix and unknown parameter vector are the same. Hence, the OMC vector *L* is the only factor that affects the final positioning result.

Four CORS stations, named P301, P171, P279 and P546, are chosen as regional reference stations for satellite clock bias estimation. Five other stations are chosen as the rover for real-time PPP. Influenced by observation satellite distribution and data quality, five common visible satellites are used for calculation. The influence on the positioning performance with the satellite clock bias estimation is analyzed.

**Figure 3:**
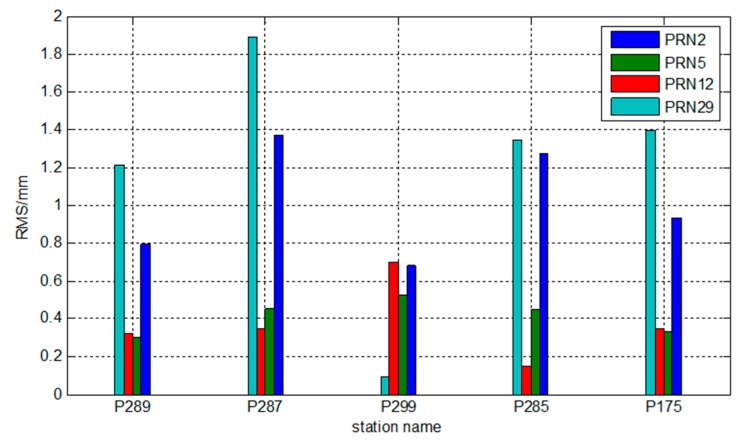
Statistical RMS values of the OMC vector of observation equations in different stations.

The OMC vector *L* of observation equation for the regional reference network by using the traditional PPP method and the PCCO method on real-time PPP is calculated. The difference between the two OMC vectors is analyzed, and the root-mean-square (RMS) values are calculated for statistics. The specific results are shown in Figure 3.

Although the accuracy of broadcast ephemeris is poor (usually approximately 1–2 m for orbit precision), Figure 3 shows that the OMC vector of observation equation based on PCCO is the same in the millimeter level as traditional PPP, thus indicating that PCCO meets the request of PPP and that the residual unabsorbed errors do not affect the positioning performance in a regional area.

## 3. Real-Time PPP System Based on PCCO

**Figure 4:**
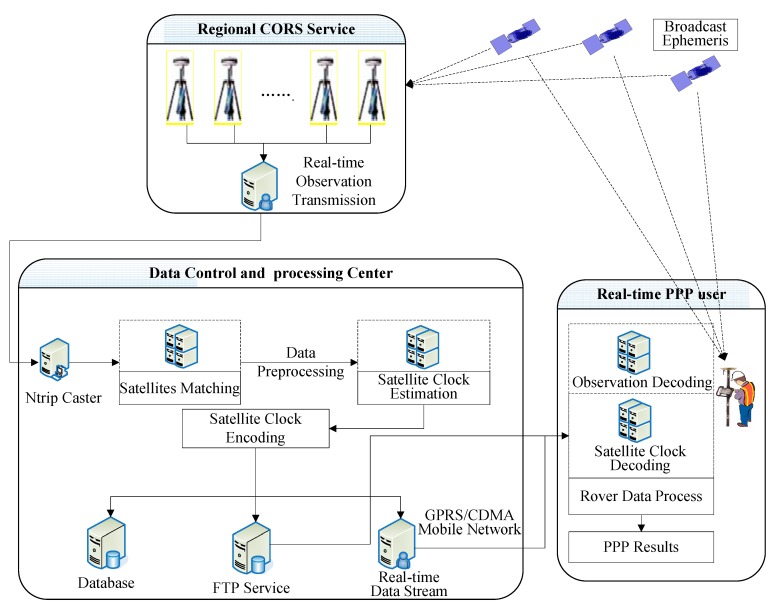
Overall structure and data stream of real-time PPP based on PCCO.

The real-time PPP system mainly includes two parts, the data control and processing center and the real-time PPP user. The data control and processing center is mainly used to calculate the coupling parameter of clock bias and orbit error (abbreviated as PCCO) and then broadcasts the PCCO to the PPP user in real time via the mobile network. The PPP user receives PCCO, GNSS observation, and broadcast ephemeris in real time and then executes PPP. The overall system structure and data flow are shown in Figure 4.

The detailed process of realization is as follows:

(1) The “regional CORS reference stations” receive GNSS observations and broadcast ephemeris.
(2) The “data control and processing center” receives observation data and the broadcast ephemeris from the regional CORS and then calculates the PCCO after data preprocessing.
(3) The PCCO is encoded, instantaneously broadcasted to the rover user by a mobile network, and uploaded to the database for post-processing users. Since the single-difference results clock the bias of a pair of satellites, the satellite clock bias is given to the rover in pairs.
(4) The PPP user receives observation data and broadcast ephemeris and obtains the clock bias coupled with orbit error followed by PPP.

The error equation of the PCCO method has been introduced in the previous section. The rover positioning error equation is expressed as follows:

(12) $$\left[\begin{array}{c} v_{1-ref}\\ v_{2-ref}\\ v_{3-ref}\\ \text{L}\\ v_{n-ref} \end{array}\right]=\left[\begin{array}{ccccccccc} \alpha_{x}^{1-ref} & \alpha_{y}^{1-ref} & \alpha_{x}^{1-ref} & M_{1-ref} & 1 & 0 & 0 & 0 & 0\\ \alpha_{x}^{2-ref} & \alpha_{y}^{2-ref} & \alpha_{z}^{2-ref} & M_{2-ref} & 0 & 1 & 0 & 0 & 0\\ \alpha_{x}^{3-ref} & \alpha_{y}^{3-ref} & \alpha_{z}^{3-ref} & M_{3-ref} & 0 & 0 & 1 & 0 & 0\\ \text{L} & \text{L} & \text{L} & \text{L} & \text{L} & \text{L} & \text{L} & \text{L} & \text{L}\\ \alpha_{x}^{n-ref} & \alpha_{y}^{n-ref} & \alpha_{z}^{n-ref} & M_{n-ref} & 0 & 0 & 0 & 0 & 1 \end{array}\right]\left[\begin{array}{c} \text{Δ}x\\ \text{Δ}y\\ \text{Δ}z\\ zpd\\ A_{IF}^{1-ref}\\ A_{IF}^{2-ref}\\ A_{IF}^{3-ref}\\ \text{L}\\ A_{IF}^{n-ref} \end{array}\right]\text{-}\left[\begin{array}{c} L_{IF}^{1-ref}\\ L_{IF}^{2-ref}\\ L_{IF}^{3-ref}\\ \text{L}\\ L_{IF}^{n-ref} \end{array}\right]$$

In the rover positioning equation, the unknown parameters contain 3D coordinates, the wet part of zenith troposphere delay, and phase ambiguity. Only the phase observations are used for positioning; thus, the equation number is n−1. In the design matrix, the first three columns are the coefficients of Δx,Δy,Δz the fourth column is the coefficient of the wet part of tropospheric delay; and the other columns are the coefficients of the single difference ambiguities between satellites. The algorithm is used to develop our own PPP software, named SEUP3, which could receive PCCO and decode it for PPP.

Compared with traditional real-time PPP, the advantages of this system are as follows:

(1) In this system, satellite clock bias is coupled with orbit error as an estimation parameter. Therefore, precise ephemeris is no longer demanded because broadcast ephemeris is enough for real-time PPP.
(2) PCCO can generate real-time clock bias coupled with orbit error by regional CORS and can broadcast to the rover instantaneously. PCCO has better real-time performance than the IGS real-time data stream.
(3) PCCO only needs regional CORS and does not rely on global distribution station; thus, the PCCO method is easy to implement and is suitable for engineering practice.

## 4. Experiment and Analysis

**Figure 5:**
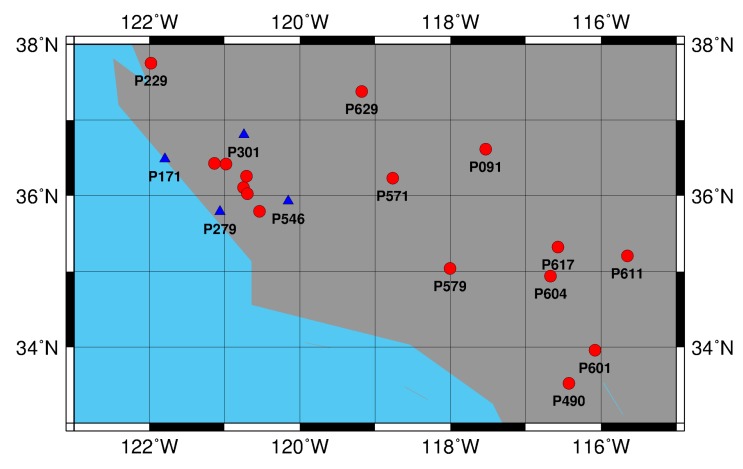
Network distribution in the experiment.

In this study, observation data from 20 stations of National Geodetic Survey (NGS) network on 8 August 2013 were used for the experiment. The data sampling rate was 15 s. The distribution of the 20 stations is shown in Figure 5. The four stations (blue triangles) were used to make the network for PCCO estimation. The lengths of the four sides of the network by four stations were 100, 110, 101 and 82 km. Another 15 stations (red circles) were selected as rovers for the PPP accuracy test. Among these stations, five were used for the experiment within the network and the others were used for the experiment outside the network. Based on the real-time PPP system designed in Section 3, the self-developed software SEUP3 is applied to compare PCCO with IGS real-time service for the rovers.

### 4.1. Experiment within the Network

**Figure 6:**
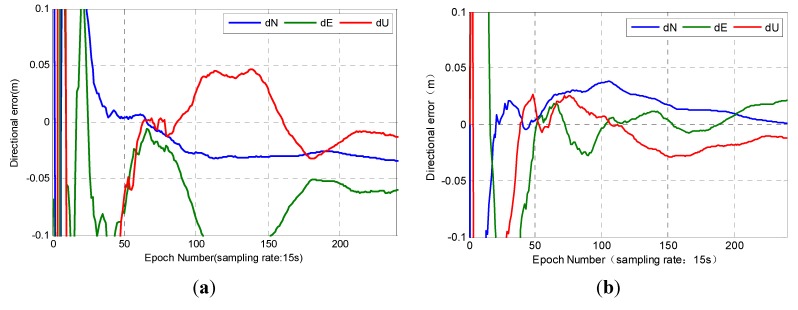
Results of station P175. (**a**) RSDI; (**b**) PCCO.

Station P175 was randomly selected as the representative of rovers within the network. We conducted real-time PPP on the basis of PCCO with regional CORS. The experimental result was compared with real-time PPP obtained by SEUP3 software on the basis of the real-time **s**treaming data from IGS (RSDI) from precision and convergence speed. We defined convergence as when the positioning error in any of the three directions becomes less than 10 cm. In addition, we calculated the RMS value of the positioning errors from the convergence epoch to the end. The results are shown in Figure 6.

Figure 6 shows that the results of PPP based on two methods are different on P175. The positioning accuracy of PCCO was significantly better than the results of RSDI. A comparison of the positioning results of P175 indicated that the convergence speed of RSDI was slower in each direction. The convergence time was 38 min, and the position error was 7.29 cm according to RSDI. The convergence time of PCCO was 9.75 min, and its position error was 3.32 cm by using PCCO. The results showed that the PCCO method was feasible in this station, which can achieve high-precision results in the U direction. PCCO can reach better results in the N and E directions than RSDI. The position accuracy increased by 54.4%, and the convergence speed increased by 74.3%.

**Figure 7:**
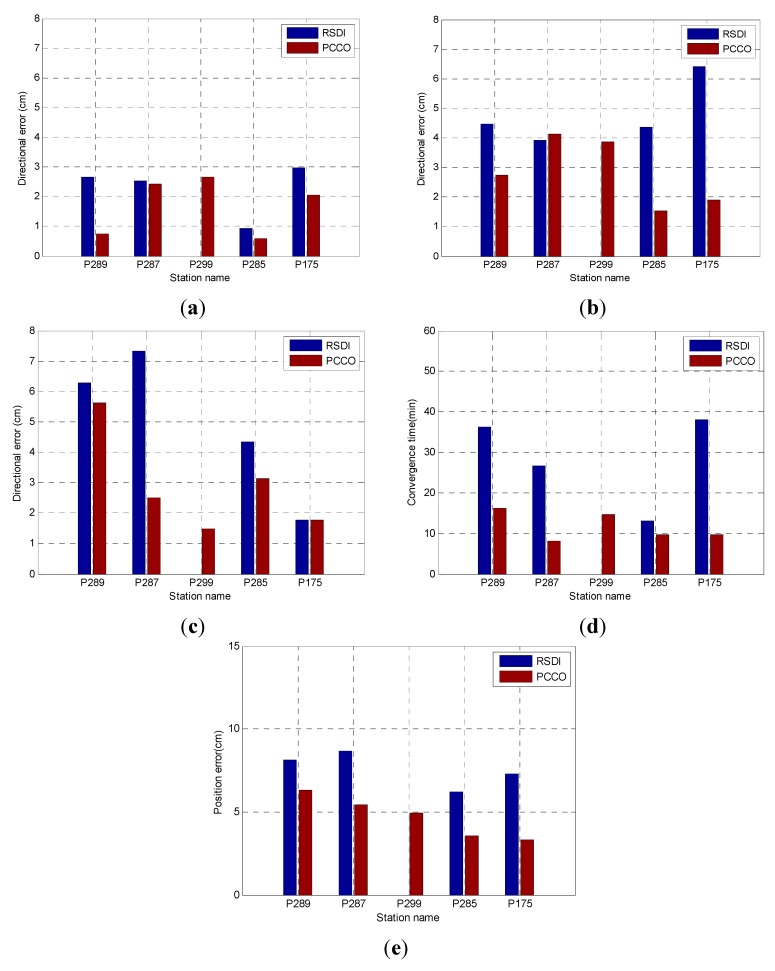
Results of the five stations within the network: (**a**) N; (**b**) E; (**c**) U; (**d**) convergence time; (**e**) position error.

The precision and convergence time of PCCO were compared with those of RSDI for five stations within the network to further analyze the effect of PCCO. The statistics of precision mainly involved the RMS of three direction errors (N, E and U) and position error. The results of the two methods are shown in Figure 7.

Figure 7 compares the positioning results of PCCO for the five stations within the network with the results of RSDI. It should be noted that, for station P299, the positioning accuracy cannot converge to achieve 10 cm within 1 h by using RSDI, so it is blank in Figure 7. The precision of the two methods could reach better than 3 cm in N direction. The precision of PCCO could be significantly improved in the E and U directions. Furthermore, the convergence speed and position precision were both improved obviously in the five stations and PCCO can achieve high precision on real-time positioning. The quantitative comparison results are shown in Table 1.

**Table 1:** RMS of positioning error and convergence time of RSDI and PCCO.

Station Name	RSDI					PCCO				
	Directional Error (cm)			Position Error (cm)	Convergence Time (min)	Directional Error (cm)			Position Error (cm)	Convergence Time (min)
	N	E	U			N	E	U		
P289	2.65	4.48	6.28	8.16	36.25	0.75	2.74	5.62	6.30	16.25
P287	2.52	3.91	7.33	8.68	26.75	2.42	4.14	2.50	5.41	8.25
P299	-	-	-	-	-	2.67	3.87	1.47	4.93	14.75
P285	0.94	4.36	4.35	6.23	13.00	0.58	1.54	3.14	3.55	9.75
P175	2.97	6.42	1.78	7.29	38.00	2.07	1.89	1.78	3.32	9.75

“-” represents a result that cannot be converged to 10 cm within 1 h.

The position precision of the five stations by PCCO was generally higher than that by RSDI and increased by 38.8% on average except P299. The convergence time was greatly shortened and improved by 61.4% on average when compared with RSDI except P299. Therefore, real-time positioning can be achieved by PCCO for stations within the network and PCCO can achieve higher precision and faster convergence speed than RSDI.

### 4.2. Experiment Outside the Network

**Figure 8:**
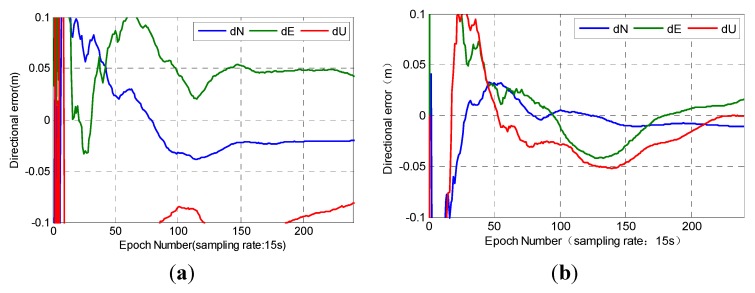
Results of station P091. (**a**) RSDI; (**b**) PCCO.

Station P091 was randomly selected as representative of rovers outside the network. The results were compared with the real-time PPP obtained by SEUP3 software on the basis of RSDI. The effect of PCCO was analyzed from the precision and convergence time, which was similar to station P175. The results are shown in Figure 8.

The distance from the network center to station P091 was approximately 308 km. RSDI and PCCO needed 46.75 and 7.5 min until the error converged to 10 cm in each direction, respectively. The position error was 4.59 cm by PCCO and 10.45 cm by RSDI after convergence. The positioning accuracy of the two methods was excellent in the N direction. The position accuracy increased by 56.1% on average, and the convergence speed increased by 83.9% on the basis of PCCO. The external station P091 was far from the network center, but its position accuracy was similar to that of the internal station. Both of the stations within and outside the network can achieve better results than RSDI. The accuracy was better than 2 cm in the N direction, which was similar with RSDI. PCCO could be applied well not only to the internal network station but also to the external station, which was 300 km away from the network.

**Figure 9:**
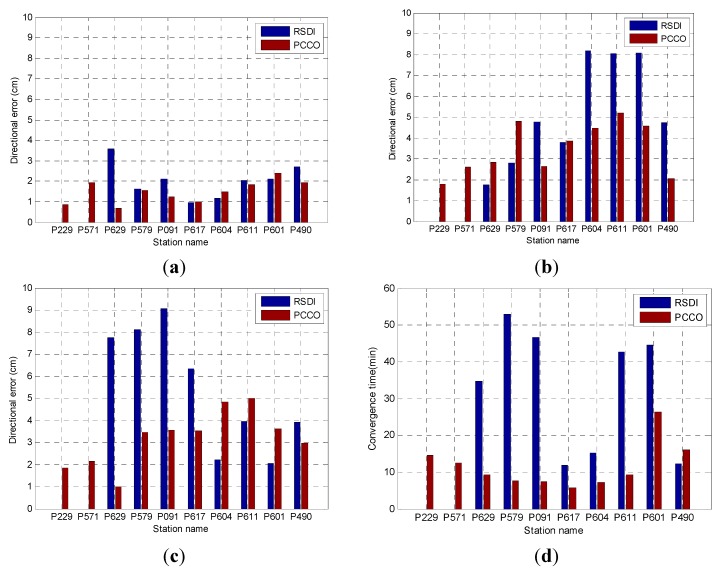

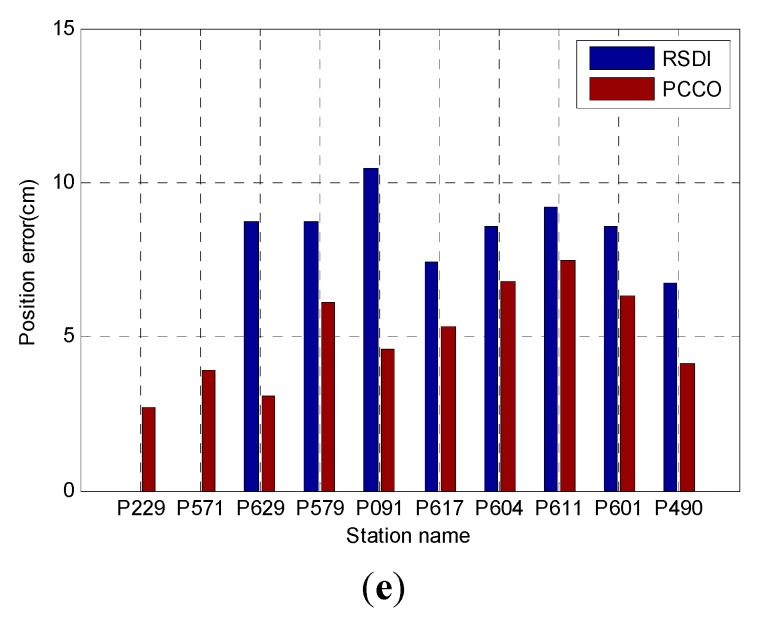
Results of stations outside the network: (**a**) N; (**b**) E; (**c**) U; (**d**) convergence time; (**e**) position error.

The precision and convergence time of 10 stations outside the network were calculated and analyzed to further test the performance of PCCO for stations outside the network. These stations were analyzed in turn according to the distance to the network center from near to far (ranging from 190 km to 511.6 km). The positioning scheme and statistical method were the same as those presented in Section 4.1. The results are shown in Figure 9. On the basis of RSDI and PCCO, the time when the errors in the N, E, and U directions converge to less than 10 cm and the RMS of positioning error within 1 h after convergence are shown in Figure 9 and Table 2. The blank represents where it cannot converge to 10 cm within 1 h.

**Table 2:** RMS of the positioning error and convergence time of RSDI and PCCO.

Station Name	Distance (km)	RSDI					PCCO				
		Directional Error (cm)			Position Error (cm)	Convergence Time (min)	Directional Error (cm)			Position Error (cm)	Convergence Time (min)
		N	E	U			N	E	U		
P229	190.0	-	-	-	-	-	0.86	1.80	1.84	2.71	14.50
P571	195.1	-	-	-	-	-	1.95	2.60	2.16	3.90	12.50
P629	200.3	3.59	1.76	7.75	8.72	34.75	0.69	2.83	1.01	3.08	9.25
P579	297.7	1.61	2.79	8.12	8.74	53.00	1.54	4.80	3.46	6.11	7.75
P091	308.0	2.10	4.77	9.06	10.45	46.75	1.24	2.63	3.55	4.59	7.50
P617	407.9	0.96	3.78	6.34	7.44	11.75	1.01	3.84	3.54	5.32	5.75
P604	413.2	1.18	8.18	2.22	8.56	15.25	1.48	4.49	4.84	6.77	7.25
P611	492.2	2.03	8.04	3.95	9.19	42.75	1.82	5.21	5.01	7.45	9.25
P601	510.6	2.13	8.07	2.06	8.60	44.50	2.38	4.56	3.63	6.30	26.25
P490	511.6	2.72	4.74	3.91	6.72	12.25	1.93	2.06	2.98	4.10	16.00

“-” represents a result that cannot be converged to 10 cm within 1 h.

Figure 9 and Table 2 show that the precision of PCCO outside the network significantly increased in the E direction and was similar to the N direction with RSDI. The accuracy of the U direction is improved for stations within about 400 km, but the positioning accuracy decreased with the increase of distance. The results of two stations (P229 and P571) cannot be converged to 10 cm within 1 h by RSDI. The convergence speed and position accuracy of all stations outside the network were significantly improved by PCCO by 65.9% and 36.1% on average in comparison with RSDI except P299 and P571, respectively.

For the rovers inside and outside the network within a certain distance, real-time PPP could be better achieved on the basis of PCCO. By using PCCO, the position accuracy increased significantly and the convergence speed improved greatly. PCCO can satisfy the cm-level accuracy requirement for stations within 500 km.

## 5. Conclusions

Satellite clock bias can absorb the projection of orbit error onto the satellite-receiver range. Therefore, an approach is proposed for the coupling estimation of clock bias and orbit error in CORS data processing center. According to the results of the proposed method, the satellite orbit from broadcast ephemeris was enough for PPP in a large-scale area (much larger than the CORS service coverage area) and precise orbit products were no longer needed. An experiment was conducted by using the NGS network observation data. The positioning results were compared with RSDI. The main conclusions are as follows:

(1) The projection of satellite orbit error was similar to the clock bias on PPP; hence, they can be coupled as one parameter for estimation purposes. During PPP by the user, the OMC vectors of GNSS equations by coupling estimation were almost the same as the traditional PPP (with a difference in millimeters). Therefore, the orbit error was availably absorbed by clock bias, whereas the residual unabsorbed orbit error did not affect the positioning in a regional area.
(2) For the stations inside and outside (with a distance less than 500 km) the network, the proposed approach performs better than RSDI. The position accuracy of the stations inside and outside the network improves by 38.8% and 36.1%, respectively, and the convergence speed improves by 61.4% and 65.9%, respectively. This new approach has the advantages of autonomy, real-time processes, and simple parameters, which is an improvement over traditional PPP. It could be an alternative solution for regional positioning service before global PPP service comes into operation.
